# Chiral Perovskite Single Crystals: Toward Promising Design and Application

**DOI:** 10.3390/ma18112635

**Published:** 2025-06-04

**Authors:** Lin Wang, Jie Ren, Hanying Li

**Affiliations:** MOE Key Laboratory of Macromolecular Synthesis and Functionalization, International Research Center for X Polymers, ZJU-YST Joint Research Center for Fundamental Science, Department of Polymer Science and Engineering, Zhejiang University, Hangzhou 310027, China; wl_zju@zju.edu.cn

**Keywords:** chiral perovskite single crystals, synthesis, chirality transfer, circularly polarized light detection, nonlinear optical response

## Abstract

Organic–inorganic hybrid halide perovskites have emerged as promising optoelectronic materials owing to their exceptional optoelectronic properties and versatile crystal structures. The introduction of chiral organic ligands into perovskite frameworks, breaking the inversion symmetry of the structure, has attracted significant attention toward chiral perovskites. Herein, the recent advances in various synthesis strategies for chiral perovskite single crystals (SCs) are systematically demonstrated. Then, we elucidate an in-depth understanding of the chirality transfer mechanisms from chiral organic ligands to perovskite inorganic frameworks. Furthermore, representative examples of chiral perovskite SC-based applications are comprehensively discussed, including circularly polarized light (CPL) photodetection, nonlinear optical (NLO) responses, and other emerging chirality-dependent applications. In the end, an outlook for future challenges and research opportunities is provided, highlighting the transformative potential of chiral perovskites in next-generation optoelectronic devices.

## 1. Introduction

In recent years, metal halide perovskites (MHPs) have emerged as a class of promising semiconductor materials for next-generation optoelectronics. While their diverse crystal structures and outstanding physical properties have fueled advancements in light-emitting diodes (LEDs) [1,2,3], coherent light sources [4,5], photodetectors [6,7,8], and photovoltaic cells [9,10,11,12], the centrosymmetric crystal structures of conventional MHPs [13,14,15,16] impose fundamental limitations for chiral photonic devices and applications. Specifically, the absence of intrinsic structural chirality in these systems severely restricts chiroptical responses and creates synthetic challenges in achieving precise control over non-centrosymmetric architectures through conventional ion substitution approaches [17].

The strategic incorporation of chiral organic ligands into perovskite lattices effectively addresses these dual challenges [18]. These chiral-induced non-centrosymmetric structures endow perovskites with unique physical properties, such as circular dichroism (CD), circularly polarized photoluminescence, nonlinear optical (NLO) responses, ferroelectricity, bulk photovoltaic effects, and chiral-induced spin selectivity (CISS) effects. The first report of chiral perovskites SC structures can be traced back to 2003 and was produced by Billing et al. [19]. Subsequent structural characterizations of other chiral perovskite single crystals (SCs) were systematically conducted in 2006 and 2013 [20,21]. Nevertheless, the intrinsic chiroptical properties of these materials remained uninvestigated. The CD characteristics of chiral perovskite thin films were first explored by Moon et al. in 2017 [22].

This discovery saw increasing research attention being paid toward chiral perovskites, focusing on their chiroptical activity and optoelectronic properties for applications in chiral optoelectronics [23,24,25,26], spintronics [27,28,29,30,31], and ferroelectrics [32,33,34]. He et al. reported the first circularly polarized luminescence-active perovskite nanocrystals (NCs) [35]. In 2018, Long et al. subsequently demonstrated spin-polarized absorption and photoluminescence in reduced-dimensional chiral perovskites [30], highlighting their potential for spin-based applications. In 2019, Chen et al. pioneered the development of circularly polarized light (CPL) photodetectors based on chiral one-dimensional (1D) perovskite thin films [36]. Ma et al. further advanced the field by realizing efficient CPL emission in chiral two-dimensional (2D) perovskite SCs [23]. Concurrently, Yang et al. reported the first 2D homochiral lead iodide perovskite ferroelectrics SCs [37], while Yuan et al. demonstrated a polarization-dependent second harmonic generation (SHG) in chiral 2D perovskite nanowires [38]. Dang et al. conducted in-depth investigations of the chiroptical properties of (*R*)- and (*S*)-MBAPbX_3_ (X = Br, I) SCs, revealing pronounced CD signals and distinct CPL emission characteristics [39].

The optoelectronic properties and device performance of chiral perovskite materials are dictated by their multiscale structural configurations. This necessitates comprehensive investigations into the origins of hierarchical chirality and further mechanistic elucidation of the structure–property relationships in these chiral systems. Dong et al. [40] and Long et al. reviewed the general optoelectronic properties of chiral perovskites [41]. Wei et al. summarized the synthetic methodologies, characterization techniques, and spin-optoelectronic and spintronic applications, presenting theoretical analysis of the chirality-related properties of chiral perovskites [42]. Dang et al. [43] and Ma et al. reviewed the crystal structures, chirality origins, the optoelectronic properties and applications of chiral perovskites [44]. The review of Ma et al. [45] and Pietropaolo et al. [46] discussed the chiral transfer mechanisms and chiral amplification mechanisms in chiral perovskites. Although chiral perovskite systems have experienced rapid research advancements, a comprehensive update of recent advances is crucial for developing chiral perovskite-based applications.

Therefore, this review focuses on the recent research progress into chiral halide perovskites in terms of synthesis methods, chirality induction mechanisms, and their optoelectronic applications. This is because chiral perovskite SCs demonstrate superior charge carrier mobility, lower defect density, and enhanced chiroptical properties compared to polycrystalline films, which results in significantly improved efficiency and stability in optoelectronic devices. Our discussion uniquely focuses on the chiral perovskite SCs, in the particular, the synthetic methods and growth mechanisms of chiral perovskite single-crystal thin films (SCTFs), as well as their applications in CPL photodetection, NLO response, and other emerging chirality-dependent applications. Our work highlights a crucial research paradigm. Furthermore, we present a forward-looking perspective on the research field, highlighting key challenges that need to be addressed and opportunities to fully harness the potential of chiral perovskite SCs in next-generation optoelectronics.

## 2. Synthesis Strategies of Chiral Perovskites

### 2.1. Chiral Perovskite Bulk SCs

Chiral perovskite SCs are usually prepared by mixing perovskite precursors together under proper conditions, including antisolvent vapor-assisted crystallization (AVC), aqueous synthesis, slow evaporation crystallization, temperature-lowering crystallization, and the inverse-temperature crystallization method (Table 1).

**Figure 1:**
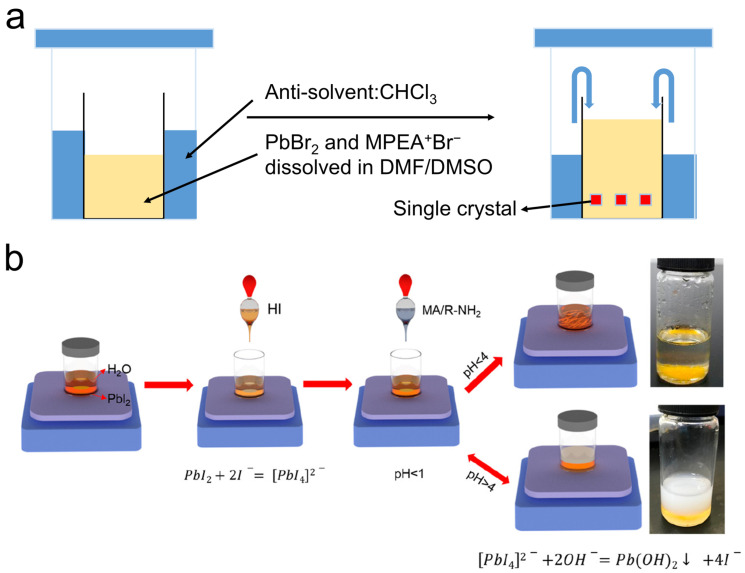
(**a**) Setup for antisolvent vapor-assisted crystallization method. (**b**) Schematic of aqueous synthesis [47]. Reproduced with permission, copyright 2019, American Chemical Society.

In the AVC approach, an antisolvent is employed to induce slow vapor diffusion into the chiral perovskite precursor solution (Figure 1a), thereby reducing the system’s solubility and facilitating the nucleation and crystallization of SCs. Yuan et al. demonstrated the growth of solvent-engineered chiral perovskite SCs using the AVC method [38], wherein a ternary solvent system comprising dimethylformamide (DMF) and dimethyl sulfoxide (DMSO) was utilized instead of pure DMF in order to optimize the optical quality of the crystals for single-crystal X-ray diffraction analysis. The solvent DMSO is directly incorporated into the perovskite crystal lattice, forming layered 2D perovskite materials in which DMSO molecules are axially coordinated with Pb^2+^ ions of the partially edge-sharing octahedra, with the chemical formula (MPEA)_1.5_PbBr_3.5_(DMSO)_0.5_.

Aqueous synthesis represents a simple and environmentally friendly approach for the synthesis of chiral organic–inorganic hybrid perovskites (OIHPs) at room temperature without extra heating. Wang et al. developed this method by employing deionized water as the solvent and precisely controlling the pH of the aqueous solution (Figure 1b) [47]. Through careful pH modulation (below 4), they successfully synthesized both 1D and 2D perovskite SCs, whereas a pH above 4 led to the formation of Pb(OH)_2_ precipitates. This strategy enabled the high-yield and reproducible synthesis of a series of chiral 2D perovskite SCs. This innovative methodology utilized deionized water as a replacement for toxic solvents (e.g., DMF/DMSO), enabling the room-temperature synthesis of 1D and 2D perovskite SCs without thermal annealing. It prevented high-temperature energy consumption and organic solvent contamination. Achieving a high product yield of 80%, this approach demonstrated the significant minimization of lead-containing byproduct generation, thereby effectively mitigating environmental contamination risks from Pb^2+^ species. The synthetic route features simplified process and efficient resource utilization, aligning with the core principles of green chemistry. However, the bulky chiral cations were predominantly distributed on the crystal surface rather than the inorganic octahedral cavities, limiting this method to the synthesis of low-dimensional perovskite SCs.

Slow evaporation crystallization, a simple and controllable SC synthesis method, has been proven effective in synthesizing chiral lead-free perovskites with high photoluminescence quantum yield and pronounced circularly polarized luminescence. He et al. employed this method by dissolving chiral organic ligands *R*-/*S*-MBA and inorganic precursors (InCl_3_ and SbCl_3_) in a mixed solvent system comprising hydrochloric acid and methanol [49]. The controlled evaporation process at 50 °C for 72 hours yielded phase-pure, millimeter-scale SCs (1.5 × 0.6 × 0.4 mm^3^) with excellent morphological uniformity. Critical to the optoelectronic performance, Sb^3+^ doping was systematically optimized to 0.55 mol%, resulting in the significant enhancement of luminescent characteristics. Single-crystal X-ray diffraction analysis unambiguously confirmed both the structural purity and chiral crystallization in the *P*1 space group, validating the successful incorporation of chiral organic moieties into the perovskite framework.

Temperature-lowering crystallization is a well-established methodology for the growth of chiral OIHP SCs, in which crystal nucleation and growth kinetics are influenced by temperature. In this method, solutions of chiral cation solution and Pb(AcO)_2_ or PbO powder are initially dissolved in an aqueous HX/H_3_PO_2_ (X = Cl, Br, I) mixed solvent system at high temperatures. As the precursor solution undergoes gradual cooling to room temperature, the solubility decreases, driving the system into a supersaturated state, which facilitates the nucleation and subsequent crystallization of perovskite SCs. Millimeter-sized perovskite SCs, such as (*R*- and *S*-*α*-PEA)PbI_3_ [36], (*S*- and *R*-MBA)_2_PbI_4_ [23], and (*R*/*S*-3AMP)PbBr_4_ [32], and centimeter-sized bismuth-based perovskite SCs (*R* and *S*-*α*-PEA)_4_Bi_2_I_10_ [48] can be easily synthesized using this method.

Three-dimensional (3D) perovskites (e.g., MAPbBr_3_) typically possess centrosymmetric crystal structures and lack chiral components, making it challenging to achieve intrinsic chirality. Chen et al. employed an inverse-temperature crystallization method to synthesize chiral 3D perovskites without using chiral A-site cations [50]. Compared to achiral MAPbBr_3_ crystals crystallized though homogeneous nucleation in pure DMF, chiral MAPbBr_3_ crystals were formed in DMF solutions containing microparticles (MPs) or nanoparticles (NPs). MPs or NPs serve as foreign nucleating agents, promoting the asymmetric nucleation of chiral 3D perovskites. Using this strategy, tiny chiral tetragonal-phase MAPbBr_3_ seeds were formed via heterogeneous nucleation. Following the “near-equilibrium autocatalytic growth” model, chiral seeds amplify their chirality through autocatalytic processes, ultimately yielding chiral crystals. Chiroptical activity emerges when MA cations adopt preferential chiral orientation ordering. Chiroptical activity originates from the chiral orientation patterns of achiral A-site cations (e.g., MA^+^, FA^+^), driven by the energy minimization principle and their interactions with [PbX_6_]^4−^ octahedra, leading to the formation of chiral supercells.

### 2.2. Chiral Perovskite SCTFs

**Figure 2:**
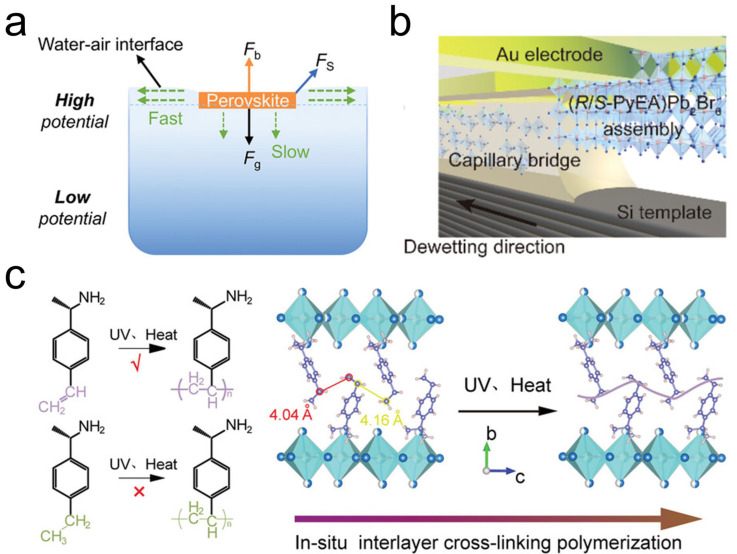
(**a**) Schematic of temperature-lowering crystallization method for growing chiral perovskite SCTFs [51]. Reproduced with permission, copyright 2025, John Wiley and Sons. (**b**) Schematic of capillary-bridge confined assembly technique [52]. Reproduced with permission, copyright 2024, American Chemical Society. (**c**) Polymerization of chiral ammonium monomer and schematic diagram of in situ polymerization reaction of interlayer chiral cations within 2D perovskites [53]. Reproduced with permission, copyright 2023, John Wiley and Sons.

Recently, Wang et al. demonstrated the growth of Dion–Jacobson (DJ)-phase chiral 2D perovskite SCTFs at the water–air interface via a nucleation-controlled crystallization method (Figure 2a) [51]. By introducing seed crystals into the refined precursor solution, the authors achieved uninterrupted crystal growth while suppressing extraneous nucleation. During the crystallization process, chiral organic cations at the water–air interface adopted a “head-down” orientation, serving as a structural template to guide the oriented growth of nucleus clusters. The enhanced chemical potential at the water–air interface, driven by surface tension, promoted anisotropic growth, ultimately yielding centimeter-sized chiral 2D perovskite SCTFs with high aspect ratios. This method enables the facile synthesis of large-sized, high-quality SCTFs through the precise control of the crystallization process.

The capillary-bridge confined assembly technique has emerged as a powerful approach for synthesizing chiral perovskite SC microwire arrays. This method utilizes an asymmetric wettability topographical template to spatially confine the chiral perovskite precursor solution (Figure 2b) [52]. As the solvent evaporates, capillary bridges undergo controlled de-wetting, inducing the nucleation and growth of highly crystalline, long-range-oriented perovskite microwire arrays on the target substrate. The method achieves a one-step process through solvent evaporation-directed oriented growth, minimizing errors introduced by traditional multi-step fabrication and enhancing process stability. The successful preparation of large-area microwire arrays on a 4-inch silicon dioxide wafer demonstrates compatibility with existing semiconductor manufacturing platforms, indicating suitability for scalable production. Additionally, the solution-based precursor processing and simplified equipment requirements align with low-cost manufacturing trends. This versatile technique is broadly applicable to chiral OIHPs with diverse compositions and dimensionalities [56,57]. Similarly, this approach has also been successfully extended to the synthesis of chiral 2D lead-free double perovskites based on Ag and Bi [58].

In another innovative approach, large-scale chiral perovskite single-crystal films were fabricated for the first time via the in situ crosslinking polymerization method [53]. The in situ crosslinking polymerization of the unsaturated vinylbenzyl groups within 2D chiral perovskite can be achieved under ultraviolet (UV) irradiation (Figure 2c). The crosslinked chiral perovskite films exhibit enhanced lattice rigidity and superior environmental stability against moisture, thermal stress, and UV irradiation compared to their uncrosslinked counterparts.

### 2.3. Chiral Perovskites Nanocrystals

Chiral perovskite NCs are typically synthesized via the post-synthetic surface functionalization of achiral perovskite cores with chiral capping ligands, in which the synergistic interplay between the achiral inorganic lattice and chiral organic moieties enables chirality transfer through ligand-induced lattice distortion and asymmetric surface interactions, thereby endowing the semiconductor nanomaterials with circularly polarized luminescence characteristics.

The synthesis of chiral perovskite NCs can be traced back to 2017, when researchers first demonstrated the assembly of chiral perovskite NCs through microwave-assisted ligand exchange [35]. In this process, achiral organic ligands capping the surface of perovskite NCs were replaced with chiral molecules, such as *R*- and *S*-1,2-diaminocyclohexane (*R*- and *S*-DACH), using CsPb(I/Br)_3_ NCs as the chiral cores. The resulting chiral structures arise from surface distortions or defects induced by the chiral aggregation of stabilizers on the NCs’ surfaces.

Further advancements include the single-step ultrasonication synthesis of colloidal cesium lead bromide (CsPbBr_3_) NCs modified with chiral *α*-octylamine, which exhibit two-photon absorption-based CPL luminescence [54]. The chiroptical properties in these systems are attributed to chiral ligand-induced asymmetric lattice distortions at the NCs’ surfaces. However, conventional purification procedures often remove chiral ligands, diminishing the CPL response. To address this, a post-synthetic chiral–ligand exchange treatment was developed to purify formamidinium lead bromide (FAPbBr_3_) NCs with chiral (*R*-/*S*-) methylbenzylammonium bromide (*R*-, *S*-MBA:Br) ligands, reactivating CPL emissions at room temperature [59].

Shi et al. reported the co-assembly of achiral perovskite NCs with chiral gels in a non-polar solvent through a supramolecular self-assembly approach [55]. Organic lipid N,N’-bis(octadecyl)-l-glutamic diamide (LGAm) and its enantiomer DGAm were used as lipids to be mixed with achiral perovskite NCs to form cogels. This approach, employing additional chiral compounds, enables the engineering of unique morphological features in chiral perovskite NCs, thereby inducing circularly polarized luminescence.

### 2.4. Chiral Perovskites Polycrystalline Thin Films

**Figure 3:**
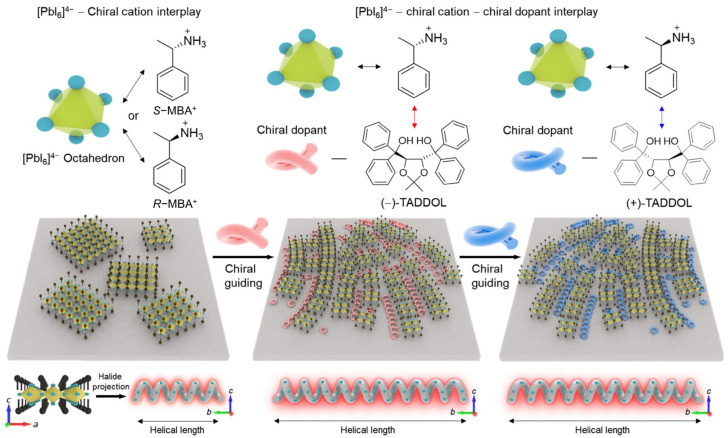
Schematic representation illustrating structural evolution from typical chiral 2D OIHPs to long-range chiral assemblies [60]. Reproduced with permission, copyright 2024, American Association for the Advancement of Science.

Recent developments include the introduction of secondary chiral dopants [(+/−)-TADDOLs] into chiral 2D OIHP thin films containing chiral organic ligand cations (*R*/*S*-MBA^+^), which induce the formation of long-range chiral assemblies and distinctive spherulitic tornado-like morphologies (Figure 3) [60]. These structures efficiently amplify the chiroptical response through a compressive strain process, wherein the chiral 2D OIHP crystals are split into discrete domains, contributing to the lattice distortion within inorganic frameworks.

### 2.5. Defect Management Strategies in Chiral Perovskites

Low defect density in chiral perovskite SCs is crucial for achieving high-performance optoelectronic devices. Reducing point defect density and improving the quality of chiral perovskite SCs are of great significance for obtaining optimal material properties and device performance [61]. Yuan et al. demonstrated an innovative strategy synergistically optimizing the lattice configuration and optoelectronic properties of 2D perovskites through chlorine-substituted chiral organic molecules and capillary-bridge assembly technology [62]. By replacing the para-hydrogen atom of phenethylamine with chlorine, this approach strengthens Cl-I halogen bonding interactions at the organic–inorganic heterointerface, significantly suppressing octahedral distortions in the inorganic layers and enhancing lattice rigidity. These structural refinements effectively inhibit electron–phonon coupling while boosting charge carrier mobility. The synthesized microwire arrays exhibit high crystallinity with pure crystallographic orientation, featuring suppressed defect states and fully eliminated grain boundaries. This efficiently mitigates non-radiative trapping and scattering of photogenerated carriers, enabling enhanced optoelectronic performance in perovskite-based devices. In another study, the passivation effect via halogen-halogen (X···X) bonding effectively suppresses the defect state density in brominated chiral perovskite thin films [63].

The rarely observed circularly polarized luminescence signals in chiral perovskite SCs may be attributed to linear polarization-induced luminescence effects and crystalline defects [64,65]. Chiral halide perovskites face challenges in the realm of blue-light circularly polarized luminescence [66]. The in situ halogenation strategy is anticipated to effectively passivate defects in chiral perovskites, enabling high-performance deep-blue circularly polarized LEDs. Yu et al. proposed an innovative in situ chlorination post-treatment strategy to enhance the performance of deep-blue perovskite LEDs [67]. By employing p-fluorocinnamoyl chloride (p-FCACl) in the anti-solvent to release chloride ions for incorporation into the perovskite lattice, this approach effectively passivates halide vacancie. The byproduct p-fluorocinnamic acid (p-FCA) concurrently mitigates Pb-halide antisite defects through dual coordination mechanisms: C=O group coordination with lead and hydroxyl-halide hydrogen bonding. Consequently, the optimized perovskite LED achieved a record external quantum efficiency (EQE) of 6.17% with stable deep-blue emission at 454 nm.

The structural defects in chiral perovskite thin films can be effectively mitigated by regulating crystallization kinetics through antisolvent engineering, which optimizes defect passivation and suppresses non-radiative recombination pathways. Yang et al. reported an antisolvent engineering approach to enhance the chiroptical properties of chiral 1D perovskites [68]. Their study revealed that high-polarity antisolvent chloroform forms strong hydrogen bonds with DMSO, effectively suppressing the formation of intermediate and secondary phases while accelerating perovskite crystallization. The rapid crystallization kinetics reduced iodine vacancy concentrations in thin films. The diminished iodine vacancy density strengthened hydrogen-bonding interactions between organic amine groups and inorganic octahedra, leading to symmetry-breaking distortion in the inorganic framework. This structural reorganization significantly amplified the chiroptical response, achieving a CD anisotropy factor of 2.86 × 10^−3^. The fabricated photodetectors exhibited exceptional responsivity, detectivity, and enhanced operational stability.

The passivation of defects in chiral perovskites can also draw on strategies from other fields [69], such as reversion heat treatment [70] and interface engineering [71]. Li et al. proposed a strategy for the self-induced double-interface modification of inverted perovskite solar cells using 5-fluoropyridine acid (FPA), aiming to solve the performance and stability problems caused by thw internal and interface defects of perovskite films [71]. Leveraging the C=O and N functional groups of FPA to chelate undercoordinated Pb^2+^/Pb clusters, this approach effectively passivates defects and suppresses carrier recombination while reinforcing the stability of the Pb-I framework to optimize crystallization quality. This strategy achieves an exceptional power conversion efficiency of 25.37% for the modified devices. Moreover, the optimized perovskite solar cells retain 93.17% of their initial efficiency after 3000 h of continuous illumination testing under a N_2_ atmosphere, demonstrating exceptional operational stability. The rational selection of synthetic protocols enables the preservation of lattice integrity while suppressing defect-mediated lattice distortions, ultimately stabilizing the structural framework [72]. For instance, introducing neutral molecules at the A-site of Bi-based 3D perovskites effectively balances charge distribution and preserves structural integrity, thereby preventing vacancy-induced structural collapse associated with Bi^3+^ incorporation [73].

Despite the significant advancements in the synthesis of various classes of chiral perovskite using synthesis methodologies, the development of straightforward and universally applicable construction strategies for the fabrication of large-scale chiral perovskite SCs with exceptional uniformity and superior crystalline quality remains a critical challenge, particularly as regards their integration into advanced optoelectronic devices and the exploration of novel functionalities. Furthermore, the question of how to optimize the magnitude of the chiroptical signal must also be carefully considered in the design of the synthetic methods of chiral perovskites.

## 3. Chirality Induction Mechanisms

### 3.1. Asymmetric Hydrogen-Bonding Interaction

**Figure 4:**
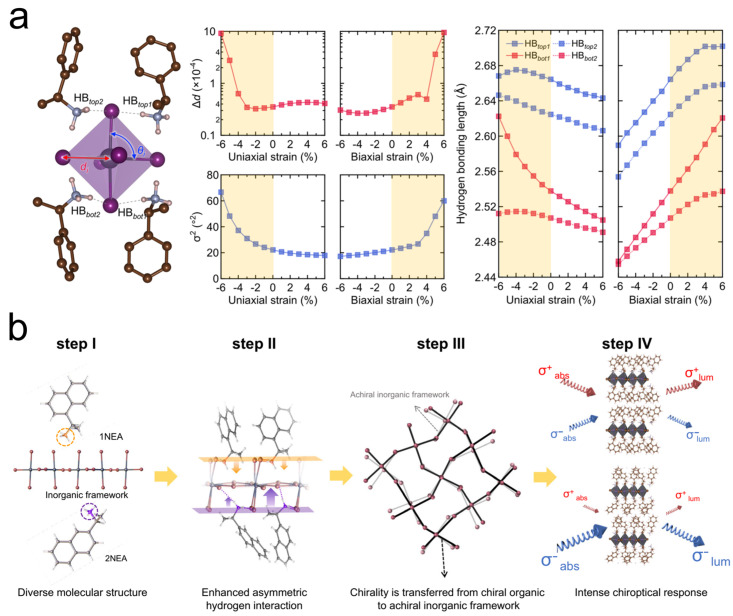
(**a**) Schematic representation of intra-octahedron distortions and hydrogen bonding. Bond length distortion, bond angle variance, and four different hydrogen bonding lengths as a function of uniaxial strain and biaxial strain [74]. Reproduced with permission, copyright 2022, Nature Publishing Group. (**b**) Schematic illustration of stepwise chirality transfer mechanism, regulating chiroptical response of chiral perovskites through distortion of inorganic layers [75]. Reproduced with permission, copyright 2023, Nature Publishing Group.

Elucidating the chirality origin mechanisms enables the rational design and targeted synthesis of chiral hybrid perovskites with tailored performance metrics for next-generation chiroptical devices. Chirality in perovskites can be achieved through the incorporation of chiral ammonium cations within the inorganic framework. The chirality transfer mechanisms in OIHPs are fundamentally determined by their crystal structures and synthetic approaches. Perovskites with intrinsically chiral crystal structures crystallize in non-centrosymmetric chiral space groups. Moon et al. reported a novel strategy for manipulating chiroptical activity through nanoconfinement-induced strain engineering [74]. Through nanoconfined growth inside anodic aluminum oxide templates, they revealed that asymmetric hydrogen-bonding interactions serve as the key factor for chiroptical phenomenon (Figure 4a). Under nanoconfinement conditions, the enhanced hydrogen-bonding interactions between chiral organic cations (MBA^+^) and the inorganic framework generate pronounced CD signals and distinct CPL emission. Micro-strain-induced conformational changes in phenyl ring stacking modulate *π*-*π* interactions and electronic coupling, thereby promoting the efficient chirality transfer from chiral organic components to the inorganic framework. Diverging from sole reliance on the chirality of the crystal structure, density functional theory (DFT) simulations were employed to assess hydrogen-bonding distances and lattice distortions, thereby validating that the electronic interaction between the chiral spacer cations and inorganic frameworks serves as the dominant mechanism for chirality transfer in perovskite systems.

To systematically elucidate the impact of hydrogen-bonding interactions on lattice distortions and coherent spin-related properties in chiral perovskites, it is essential to isolate and exclude confounding variables including molecular dimensionality and electronic structure disparities. Two structural isomers of 1-(1-naphthyl)ethylamine (1NEA) and 1-(2-naphthyl)ethylamine (2NEA) were employed as chiral spacers in 2D chiral perovskite SCs ((*R*/*S*-NEA)_2_PbBr_4_), Son et al. elucidated that the hydrogen-bonding interactions play a pivotal role in chirality transfer from chiral cations to the inorganic framework in perovskites [75]. Compared to 1NEA-based analogs, the 2NEA-incorporated chiral perovskites exhibit strengthened hydrogen-bonding networks and deeper average NH_3_^+^ cationic penetration depth, which induces more pronounced asymmetric lattice distortions in the inorganic framework. Such structural distortions significantly enhance the chirality transfer efficiency, resulting in an enhanced spin-polarized photon absorption behavior in 2NEA-incorporated chiral perovskites compared to their 1NEA-based counterparts (Figure 4b). Furthermore, the 2NEA-incorporated perovskites exhibit enhanced thermal and environmental stability, with first-principles calculations verifying their lower formation energy.

### 3.2. Interfacial Interaction

The chiroptical activity of perovskite materials can be generated through the interfacial interactions between chiral capping molecules and the achiral perovskite core. This chiral induction mechanism has been extensively investigated in chiral perovskite NCs. He et al. developed a post-synthetic ligand exchange approach to synthesize chiral perovskite NCs (CsPb(I/Br)_3_), wherein the achiral oleylamine (OA) surface ligands aggregated on the surface of the inorganic CsPb(I/Br)_3_ NCs were replaced with chiral 1,2-diaminocyclohexane (DACH) molecules [35]. The study revealed ligand concentration dependence in both chiroptical responses (mirror-symmetric CD signatures) and their underlying mechanisms. When excess DACH is employed for NC surface capping, the resulting chiroptical properties are primarily aggregation-induced, arising from the chiral packing arrangement of DACH molecules on the surface. Conversely, when minimal DACH is used for capping, the chiral optical behavior of the NCs is mainly governed by surface distortion (or defect-mediated) effects and interfacial electronic coupling mechanisms. Following DACH removal, the NCs retain residual CD signals, demonstrating that chiral configurations can be stabilized through surface distortion and/or defect engineering in the perovskite lattice.

**Figure 5:**
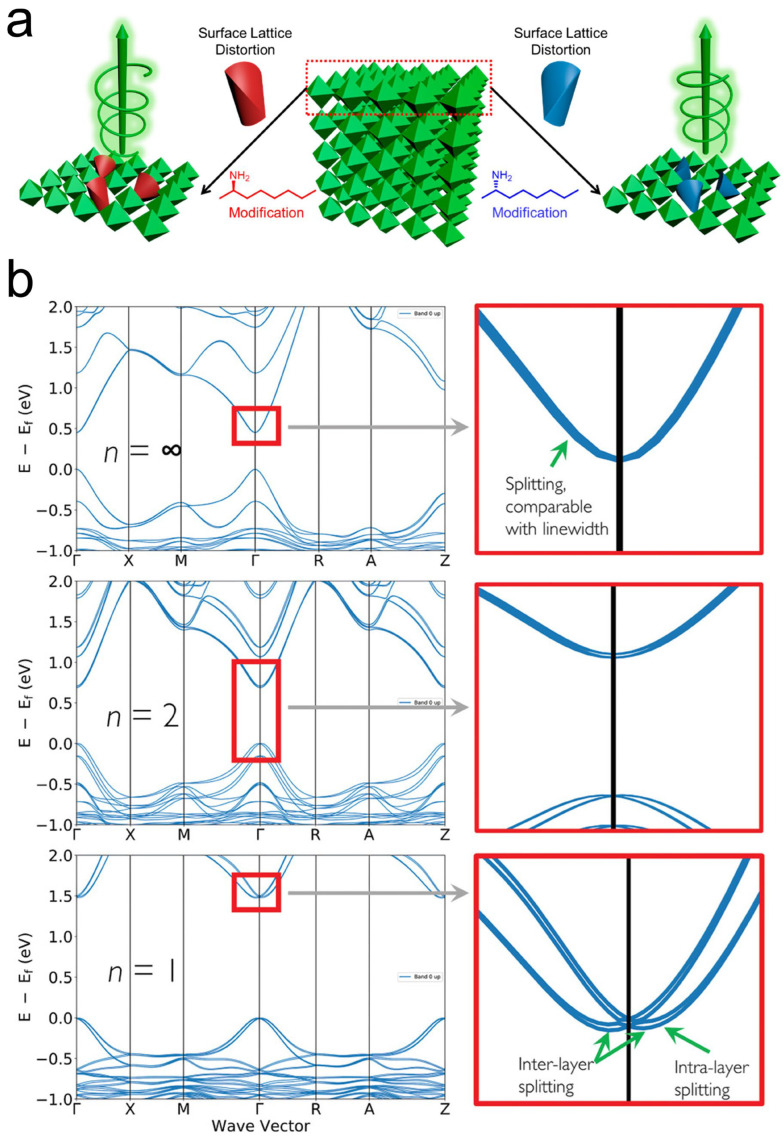
(**a**) Chiral surface distortion chiral perovskite CsPbBr_3_ [54]. Reproduced with permission, copyright 2019, American Chemical Society. (**b**) Electronic structure of (from top to bottom) perovskites MAPbI_3_, (BA)_2_(MA)Pb_2_I_7_, and (BA)_2_PbI_4_ [76]. Reproduced with permission, copyright 2021, John Wiley and Sons.

Chen et al. modified CsPbBr_3_ NCs with chiral ligands (*R*/*S*-α-octylamine) and achieved upconverted circularly polarized luminescence based on two-photon absorption, representing the first demonstration of this phenomenon in perovskite materials [54]. The mechanism of chirality generation is mainly attributed to the inducing effect of chiral ligands (such as *R*- or *S*-α -octylamine) on the surface of inorganic perovskite CsPbBr_3_ NCs. Through specific binding between the ligands and surface Br-rich defect sites, the chiral molecules induce asymmetric lattice distortion at the NC’s surface, thereby transferring chirality to the NCs and imparting circularly polarized luminescence characteristics (Figure 5a).

### 3.3. Rashba–Dresselhaus Splitting

The traditional view holds that chiral organic molecules break the symmetry of the inorganic layer through the "chiral transfer" mechanism, thereby generating CD and CPL signals. However, Pham et al. pointed out that this mechanism is not the only explanation, as achiral materials (such as monolayer MoS_2_) can also exhibit similar CD and CPL responses [76]. The observed CD and circularly polarized luminescence signals in achiral two-dimensional Ruddlesden–Popper perovskites (BA)_2_(MA)*_n_*_−1_PbnI_3*n*+1_ (where MA = methylammonium and BA = butylammonium) originate from the Rashba–Dresselhaus spin–orbit coupling (SOC) effect. The study reveals that single-layer inorganic quantum wells (*n* = 1) exhibit significant CD (≈100 mdeg) and CPL (≈4.8%) signals, which rapidly diminish with the increasing inorganic layer number (*n*). Through DFT calculations and experimental verification, the authors demonstrate that this effect originates from strong SOC, induced by lattice distortion at the organic/inorganic interface (Figure 5b). Furthermore, magnetic CD spectroscopy estimates Rashba–Dresselhaus effective magnetic fields of ≈600 mT and ≈50 mT for *n* = 1 and *n* = 2, respectively. These measurements provide further evidence regarding the correlation between SOC strength and the degree of structural distortion in the perovskite lattice. These findings offer novel design principles for Rashba–Dresselhaus effect-based spintronic devices.

Although significant advancements have been made in terms of elucidating chirality transfer mechanisms in chiral perovskites, research in this field remains in its nascent stages. Given the pivotal role of chirality in enhancing the performance of chiral perovskite-based optoelectronic devices, such as CPL photodetectors and spintronic applications, a deeper understanding of the origin of induced chirality and the underlying mechanisms of chirality transfer is imperative.

## 4. Applications in Chiral Optics and Photonic Devices

### 4.1. Chiral Perovskite SC-Based CPL Photodetector

Leveraging the strong CPL-selective absorption properties, CPL photodetectors based on 1D chiral perovskite thin films, specifically (*R*- and *S*-*α*-PEA)PbI_3_, were first implemented in direct CPL photodetection, achieving an anisotropy factor of responsivity (*g*_res_) of 0.1 [36], thereby establishing a promising platform for direct CPL detection. Inspired by this progress, various engineering strategies have been developed to enhance the sensitivity of CPL photodetectors. For instance, photodetectors based on chiral quasi-two-dimensional perovskite [(*R*)-*β*-MPA]_2_MAPb_2_I_7_ SCs exhibit improved sensitivity to CPL [77]. Additionally, the built-in electric field at the interface of a [(*R*)-*β*-MPA]_2_MAPb_2_I_7_/MAPbI_3_ heterostructure crystal facilitated efficient charge transfer and chirality transfer, resulting in the significant amplification of circular polarization sensitivity under zero-bias conditions [78]. Dimensionality engineering enables a controlled phase transition from 1D chiral perovskite nanowires to well-defined 2D layered structures, yielding remarkable improvements in both charge carrier transport and CPL detection capabilities [79]. Furthermore, dynamic crystal reconstruction in chiral 2D perovskites (*R*/*S*-MBA)_2_PbI_4_ enabled the remarkable enhancement of chiroptical activity [60]. A self-powered CPL detector was fabricated for highly sensitive CPL photodetection, achieving an impressive *g*_res_ of 1.16.

**Figure 6:**
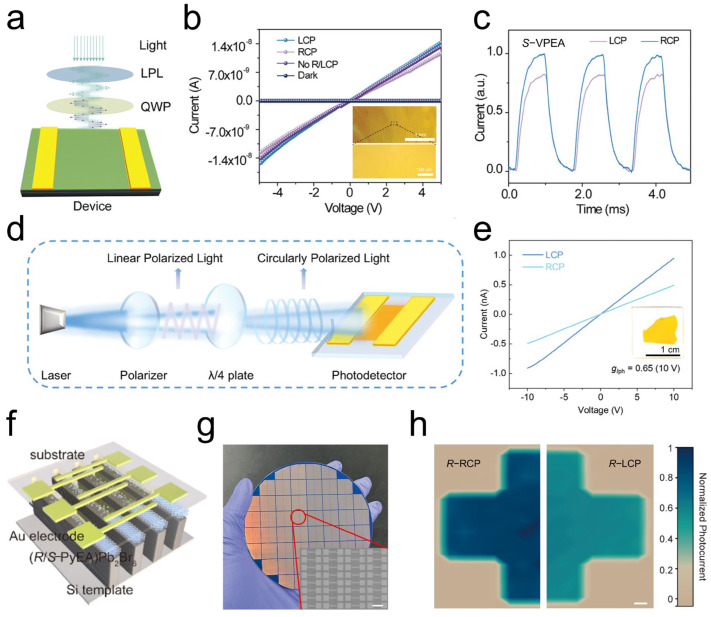
(**a**) Schematic illustration of CPL photodetector. (**b**) Photocurrent performance of crosslinked perovskite devices under dark, unpolarized light, and CPL illumination conditions. (**c**) Photocurrent of devices under CPL illumination [53]. Reproduced with permission, copyright 2023, John Wiley and Sons. (**d**) Schematic illustration of measurement of SCTF device. (**e**) *I*-*V* curves of SCTF device under CPL illumination [51]. (Reproduced with permission, copyright 2025, John Wiley and Sons). (**f**) Schematic illustration of device structure of chiral perovskite microwire array-based photodetector. (**g**) Photo of microwire arrays fabricated on a 4 in. silicon dioxide wafer. (**h**) Image of cross-shaped pattern for perovskite array device under CPL illumination. Scale bars: (**g**) 100 μm; (**h**) 200 μm [52]. Reproduced with permission, copyright 2024, American Chemical Society.

Despite these advancements, the inherent brittleness of perovskite SCs poses challenges for the development of flexible CPL photodetectors with robust mechanical stability. To address this, Zhao et al. constructed flexible CPL photodetectors based on polymerized chiral perovskite single-crystal films [53], which exhibited excellent mechanical stability due to enhanced crystallinity and pure crystallographic orientation, achieving a maximum anisotropy factor of 0.22 (Figure 6a–c). More recently, Wang et al. reported high-sensitivity CPL photodetectors based on novel DJ-phase chiral 2D perovskite SCTFs, which displayed a sensitive response to CPL with an impressively high *g_I_*_ph_ of 0.65 (Figure 6d,e) [51]. Compared to solution-processed polycrystalline thin-film devices, the enhanced long-range ordering in single-crystalline nanowire arrays significantly improves both sensitivity and stability for CPL detection. Liu et al. employed a micropillar template-assisted capillary-bridge rise method to fabricate chiral perovskite (*R* and *S*-MBA)_2_PbI_4_ nanowire arrays with exceptional phase purity and alignment uniformity [57]. The geometric alignment of nanowires increases the active area while improving device uniformity and reproducibility. The detector demonstrates exceptional optoelectronic performance, including a high on/off ratio of 1.8 × 10^4^, a responsivity of 1.4 A W^−1^, and an outstanding photocurrent anisotropy factor of 0.24, enabling efficient discrimination between left- and right-handed CPL.

Given the versatility of polarimeters in determining polarization ellipse parameters, full Stokes photodetectors based on chiral perovskites hold significant potential for next-generation photonic applications [80]. Zhao et al. pioneered the development of Stokes parameter photodetectors using chiral 2D perovskite single-crystalline nanowire arrays for arbitrary polarized light detection [56], where excellent Stokes parameter detection was achieved by correlation photocurrents under different polarization states. Ma et al. reported an all-Stokes polarimeter based on chiral 2D perovskite SCs (*S*- and *R*-MBA)_2_PbI_4_ [80]. By leveraging the intrinsic chirality of the material (for circular polarization discrimination) and its strong optical anisotropy (for linear polarization discrimination), the functionalities of polarizers, waveplates, and photodetectors were integrated together, enabling the development of a compact and low-cost miniaturized polarimeter. Under 512 nm illumination, the as-fabricated Stokes polarimeter exhibited a responsivity of 0.136 A W^−1^ and a specific detectivity of 1.2 × 10^10^ Jones. It demonstrated the capability to measure all Stokes parameters with average errors of 11% for S_1_, 7.5% for S_2_, and 26% for S_3_.

In recent years, research on chiral 2D perovskites has predominantly focused on CPL detection within the visible spectral range, while their UV photoresponse remains substantially underexplored. Zhang et al. reported the solution-grown heteroepitaxy of chiral perovskite [(*R*)-MPA]_2_PbCl_4_ SCs on silicon substrates, forming a perovskite/Si heterojunction [81]. A built-in electric field at the heterointerface promoted the efficient separation and transport of photogenerated carriers, coupled with chirality transfer, which dramatically amplified the CPL sensitivity in a solar-blind UV region. Gu et al. synthesized chiral 2D perovskite SCs *R*/*S*-(BrBA)_2_PbBr_4_, where the introduction of bromobutylammonium chiral spacers generated significant distortion in the inorganic framework [82]. Photodetectors based on *R*-(BrBA)_2_PbBr_4_ and *S*-(BrBA)_2_PbBr_4_ with vertical electrode configuration demonstrates pronounced CPL discrimination capability under 405 nm illumination, exhibiting *g_I_*_ph_ of 0.11 and 0.14, respectively. Owing to the unique semiconductor properties, the X-ray detector showed excellent X-ray detection performance with an X-ray sensitivity of 531.33 μC Gy^−1^ cm^−2^ at a bias voltage of 20 V and a detection limit of less than 100 nGy s^−1^, demonstrating the potential of chiral perovskites for use in medical imaging.

Beyond UV and visible regions, CPL photodetection in the near-infrared (NIR) region remains relatively unexplored. Peng et al. reported the first visible–NIR (vis−NIR) dual-modal CPL photodetector based on 2D chiral perovskite (*R*-BPEA)_2_PbI_4_ bulk SCs, achieving high-performance vis−NIR dual-modal CPL-sensitive detection with a *g_I_*_ph_ exceeding 0.1 [83].

Compared to low-dimensional perovskites, 3D perovskites offer advantages such as reduced exciton binding energies and superior CD absorption properties. Notably, Bai et al. synthesized 3D chiral perovskite (*R*/*S*-PyEA)Pb_2_Br_6_ microwire arrays [52], with chiral organic cations occupying the B-site of the perovskite structure, marking a significant breakthrough in the development of integrated CPL photodetectors (Figure 6f–h). Subsequently, Yang et al. synthesized chiral 3D lead halide perovskite SCs R/S [DMA]PbCl_3_, achieving excellent CPL photodetection with a *g_I_*_ph_ of 0.296 [84].

### 4.2. NLO Response of Chiral Perovskite SCs

**Figure 7:**
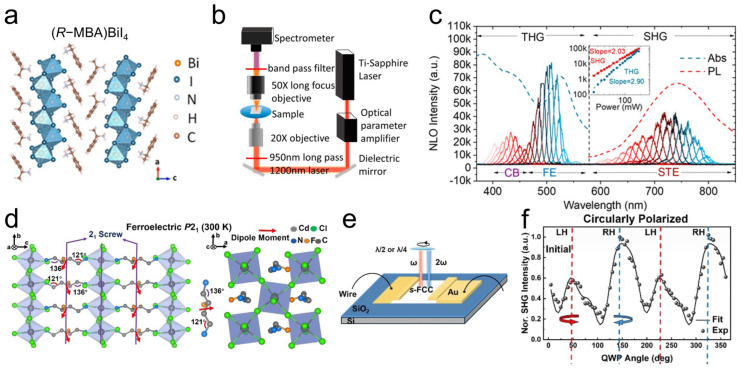
(**a**) Crystallographic structure of chiral hybrid bismuth halides. (**b**) NLO microscopic measurement setup. (**c**) Wavelength dependence of SHG and THG intensities for chiral hybrid bismuth halides film [85]. Reproduced with permission, copyright 2021, American Chemical Society. (**d**) Side view and top view of chiral ferroelectric-phase perovskite (300 K). (**e**) Schematic illustration of device configuration of electrically controllable SHG-CD. (**f**) SHG signal under CPL illumination as function of zero electrical bias [92]. Reproduced with permission, copyright 2024, American Chemical Society.

Chiral perovskites, characterized by their intrinsic non-centrosymmetric crystal structures, have emerged as highly promising materials for NLO applications. Significant research efforts have been directed toward understanding and optimizing the second- and third-order NLO properties of chiral 1D perovskites. Yao et al. reported a class of chiral hybrid bismuth halides, exhibiting resonant-enhanced SHG and third-harmonic generation (THG) performance across a broad wavelength range (400–800 nm) (Figure 7a–c) [85]. More recently, Cheng et al. reported a novel resonant channel for boosting the second-order optical nonlinearity in lead halide perovskite (C_7_H_10_N)PbBr_3_, achieving a remarkably high effective NLO coefficient of 40.2 pm V^−1^ through the deep charge transfer exciton resonance [86].

Compared to polycrystalline thin films, single-crystalline microwires exhibit lower defect density, stronger optical confinement effect, and longer carrier diffusion length, thereby significantly improving NLO performance [87,88]. Zhao et al. reported a capillary-bridge confined assembly strategy for fabricating chiral layered metal–halide perovskite *R*-, *S*-, and *rac*-ClPEA_2_PbI_4_ (ClPEA = 1-(4-chlorophenyl)ethylamine single-crystalline microwire arrays [89]. By incorporating chiral organic cations (*R*-/*S*-1-(4-chlorophenyl)ethylammonium) to break the inversion symmetry in the perovskite crystal structure, enabling exceptional second-order NLO properties, the fabricated microwire arrays demonstrated linear polarization-dependent SHG and two-photon-excited fluorescence. Due to the optical confinement and suppressed optical scattering, the SHG conversion efficiency of the prepared microline array is 3 to 5 times higher than that of the polycrystalline film counterparts.

In the periodic table, tin and lead belong to the same group. However, Sn^2+^ exhibits stereochemically active 5s^2^ lone pair electron (SCALP), which can induce stronger structural distortions and may enhance NLO performance. Tao et al. reported the first tin (II)-based chiral hybrid perovskites, (*R*/*S*-*α*-PEA)SnX_3_ (X = Cl, Br), which feature a highly distorted 1D chain structure with severely distorted [SnX_6_] octahedra [90]. The [SnX_6_] octahedra exhibit pronounced distortion due to the SCALP effect, far surpassing those of isostructural analogs. This material demonstrates remarkable SHG responses, with (*S*-*α*-PEA)SnBr_3_ showing 1.4 times the SHG intensity of *α*-quartz. It simultaneously features a broad transparency window (visible to near-infrared) and high polarization anisotropy, highlighting the great potential of Sn^2+^-based materials for NLO applications.

The efficiency of SHG signals can differ when a circularly polarized laser propagates through chiral crystals at different velocities, a phenomenon known as SHG circular dichroism (SHG-CD). The early research on the distinguished SHG-CD properties in chiral lead halide perovskite nanowires was first reported by Yuan et al. [38]. Subsequent studies by Fu et al. explored the SHG-CD effects in chiral 1D [(*R*/*S*)-3-aminopiperidine]PbI_4_ bulk SCs, achieving an anisotropy factor of 0.21 when pumped at 1064 nm [91]. Most recently, a chiral ferroelectric 2D hybrid perovskite *S*-(FBDA)CdCl_4_ (Figure 7d) was synthesized with electrically switchable SHG-CD based on multipolar effects (Figure 7e,f) [92]. The saturated polarization (*P*s) of this material, determined by the charge point model, was assessed to be 1.67 μC/cm^2^, comparable to other reported ferroelectric OIHPs.

### 4.3. Other Emerging Chirality-Associated Optoelectronics

Although lead halide perovskites exhibit high sensitivity and low detection limits in X-ray detection, their practical applications are hindered by toxicity concerns and the requirement for high operational voltages. Moreover, the promising application of chiral perovskite materials for X-ray detection remains largely untapped. Bismuth-based perovskites (e.g., Cs_2_AgBiBr_6_) require external electric fields to drive charge separation, leading to high energy consumption and complex circuit design. You et al. reported the first self-powered X-ray detector based on lead-free chiral hybrid perovskites ((*R*/*S*-PPA)_2_BiI_5_) [93]. By incorporating chiral organic cations (*R*/*S*-1-phenylpropylamine), they constructed a zero-dimensional polar structure with spontaneous polarization (23.83 μC cm^−2^). Leveraging the bulk photovoltaic effect, the built-in electric field (photovoltage: 0.63 V) generated by spontaneous polarization drives efficient charge carrier separation. The device achieves a sensitivity of 31 μC Gy^−1^ cm^−2^ and a detection limit of 270 nGy s^−1^ at zero bias, thereby addressing the critical challenge of lead-free perovskites in self-powered detection.

Isotropic radioluminescence emissions from conventional scintillators inevitably cause inter-pixel optical crosstalk, thereby degrading the spatial resolution in imaging applications. Li et al. demonstrated the existence of a new class of chiral perovskite scintillators, (*R*-3AP)PbBr_3_Cl·H_2_O and (*S*-3AP)PbBr_3_Cl·H_2_O, which exhibit a unique property of circularly polarized radioluminescence [94]. Through the strategic of left- and right-handed perovskite crystals, this material can directionally regulate the radioluminescence propagation. This significantly reduces the optical crosstalk between adjacent pixels, thereby improving the boundary resolution of X-ray imaging.

Focusing on the unique properties and applications of chiral perovskite SCs in optics and spintronics, Song et al. reported the ambient atmosphere growth of chiral perovskite (*R*/*S*-MBA)_4_Cu_4_I_8_·2H_2_O SCs via a slow evaporation method, circumventing the complexity of traditional inert-atmosphere synthesis [95]. The crystals exhibit outstanding circularly polarized luminescence characteristics with a luminescence dissymmetry factor (*g*_lum_) of 1.5 × 10^−3^ and a photoluminescence quantum yield exceeding 21%. Successful application in UV-pumped circularly polarized LEDs shows high optical selectivity, demonstrating their promising potential for advanced applications in 3D displays, spintronics, and anti-counterfeiting technologies.

**Figure 8:**
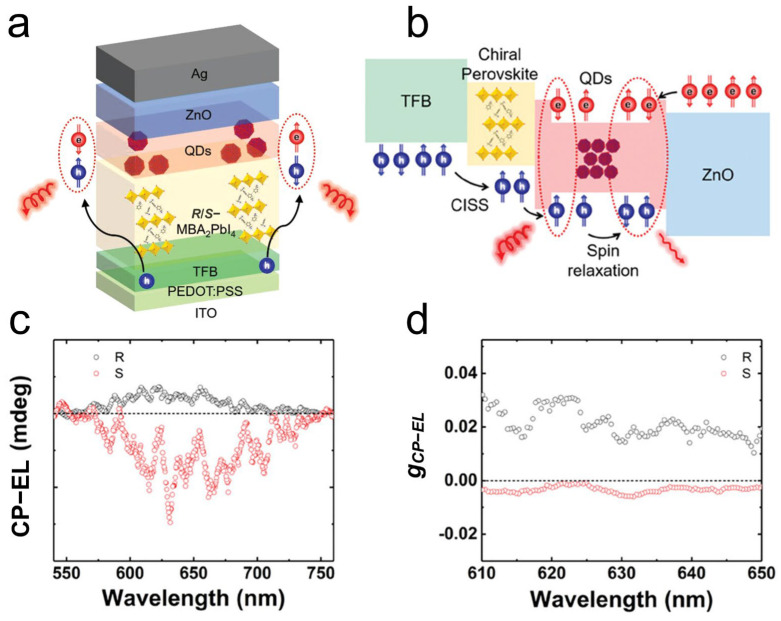
(**a**) Schematic illustration of CPEL emission for spin-quantum dots light-emitting diode (spin-QLED). (**b**) Schematic illustration of CP-EL emission in the spin-QLED. CP-EL degrees as function of (**c**) wavelength and (**d**) related asymmetric factor (*g*_CP-EL_) for devices [96]. Reproduced with permission, copyright 2023, John Wiley and Sons.

Most recently, Wang et al. demonstrated a spin LED based on chiral perovskite heterostructure films. This was fabricated by integrating 2D chiral perovskites with CdSe/ZnS quantum dots (Figure 8a) [96]. The 2D chiral perovskite layer served as a chiral-induced spin selectivity (CISS) layer, enabling spin-dependent carrier injection into the quantum dots (Figure 8b). This innovative design achieved high-performance spin LED operation at room temperature without an external magnetic field, exhibiting a circularly polarized electroluminescence (CPEL) asymmetric factor of 1.6 × 10^−2^ (Figure 8c,d). This represents a significant advancement in the field of spintronics and chiral optoelectronics.

Ferroelectricity can arise from spontaneous polarization through a nonzero electric dipole moment in the absence of an external electric field. The piezoelectric effect requires the absence of inversion symmetry in the crystal’s symmetry elements. The breaking of space inversion symmetry is crucial for ferroelectrics and piezoelectric. According to the Neumann–Curie principle governing symmetry–property relationships, chiral perovskite systems are expected to exhibit intrinsic ferroelectric and piezoelectric properties when crystallized in polar crystallographic point groups (*C*_1_, *C*_2_, *C*_3_, *C*_4_, *C*_6_). Guo et al. synthesized chiral 2D perovskites through an in situ reaction under acidic conditions [97]. By introducing chiral organic amines, they endowed the material with a non-centrosymmetric crystal structure, achieving piezoelectricity and multi-axial ferroelectricity. To address the issue of the poor stability of perovskites in water, they produced the material with polymers to form a flexible composite film, enabling prominent underwater ultrasound detection performance.

Chiral perovskite materials may possess untapped potential for emerging multifunctional applications [98]. Beyond their intriguing optical and electronic properties, the mechanical characteristics of chiral materials have garnered considerable attention for their potential in perovskite solar cells. The high structural tunability of OIHPs has enabled the engineering of chiral perovskite interfaces between the perovskite absorber layer and the electron–transport layer (ETL) [99]. The incorporation of such chiral interlayers has been shown to enhance the chemical stability and charge transfer efficiency of the resulting devices. The resultant device exhibits excellent tolerance to thermal cycling, damp heat, and prolonged light continuous, thereby addressing critical challenges in perovskite solar cell durability and performance.

## 5. Summary and Outlook

In this review, we focus on the recent progress in chiral perovskite materials, encompassing innovative synthesis strategies, chirality introduction mechanisms, and their applications in chiral optics and photonic devices. SCs serve as an indispensable platform for elucidating the intrinsic properties of these materials while also laying the groundwork for advanced optoelectronic applications. This review focuses on the synthesis methods, growth mechanisms, and photoelectric applications of chiral perovskite SCs and discusses the influence of asymmetric hydrogen bonding on the chiroptical properties. Chiral perovskites, featuring structural versatility and unique chiroptical properties, have emerged as a promising class of semiconductor for next-generation chiral optoelectronics and photonic technologies. Although significant progress has been made in this field, there are still many challenges that remain to be addressed.

First, while a variety of synthesis strategies have been developed for chiral perovskites, the available materials remain relatively limited. Expanding this diversity is crucial for transformative device applications. In terms of material design, it is imperative to implement theoretical predictions of novel chiral perovskite SCs with remarkable optoelectronic properties. From the perspective of structure dimensions, developing chiral 3D perovskite SCs is worthwhile. However, the incorporation of bulky chiral ligands into 3D perovskite lattices is hindered by steric constraints [52]. The controllable synthesis lies in screening chiral cations with appropriate tolerance factors [100,101]. Consequently, when the introduction of bulky chiral cations is required, employing an alkali metal framework with larger lattice parameters is an effective strategy to preserve the 3D structure [102]. Moreover, the rational incorporation of divalent chiral organic cations enables the design of chiral 3D hybrid perovskites (A′M_2_X_6_) with expanded [PbX_6_]^4−^ octahedral frameworks [103]. This approach enables the accommodation of bulkier chiral organic cations without compromising the topological stability of the crystal structure, while retaining the intrinsic strong SOC and superior charge-carrier transport properties characteristic of lead-based perovskites. For synthesis methods, the controlled synthesis of 3D chiral perovskites can be achieved through the non-covalent chiral templating strategy [84]. The development of scalable, environmentally benign, and uniform SCTF fabrication methods is imperative to advance their practical implementation. The surface quality of chiral perovskite SCs can be further optimized by minimizing their air exposure and residual solvent. Amphiphilic long-chain organic amines can be introduced into the precursor solution, which can spontaneously remove the residual solution and form a hydrophobic crystal surface [104]. The core of the growth of chiral perovskite SCs lies in the chiral transfer mechanism and the control of crystallization kinetics. The use of sulfonate ligands as additives enables effective nucleation suppression and controlled growth kinetics, thereby facilitating the growth of high-quality perovskite SCs with reduced defect densities [61].

Second, the research on chirality transfer mechanism in chiral perovskites is still at an early development stage, further studies should focus on elucidating the detailed amplification mechanisms of CPL sensitivity. While chiral transfer from organic ligands to perovskite lattices has been demonstrated, the precise definition and mechanistic discussion of chiroptical retention and chiral transfer efficiency in chiral perovskites require further systematic investigation. Each characteristic peak of the CD spectrum can be clearly attributed to a different exciton transition. Deconvolution methods (e.g., Gaussian peak fitting) can unveil underlying physical phenomena for excitonic transitions in chiral perovskites. The *π*-electrons of the organic spacer layer exhibit potential coupling effects with the *p*-orbitals of iodide (I^−^) from the inorganic framework [105]. Therefore, precisely modulating the electronic interactions between chiral organic molecules and the achiral inorganic lattice could further amplify the perovskite’s chiroptical response and strong anisotropic photophysical properties. Furthermore, enhancing chiral distortion through the halogen mixing strategy can significantly boost the chiroptical activity of perovskites [106].

Third, most CPL photodetectors rely on direct CD absorption for CPL detection, resulting in wavelength-dependent device characteristics and a limited NIR response due to the wide bandgap of low-dimensional chiral perovskites. Innovations in material and device structures such as heterojunctions are necessary to achieve panchromatic and broad-optical-bandwidth CPL detection, which is crucial for commercial applications. Moreover, while current efforts focus on optimizing individual devices, integrating chiral perovskite SCs into functional device arrays remains largely unexplored. Integrating chiral perovskite SCs into sophisticated optoelectronic devices can be concerned with commercial applications, such as optical communication, spintronic devices, and chiroptical sensors. To enhance device performance and reliability in practical applications, it is critical to precisely control the crystal structure, morphology, dimensions, and the directional arrangement of chiral perovskites. It can also improve their electron transport properties.

Finally, most NLO-related studies have focused on the harmonics generated by circularly polarized fundamental lasers, with limited systematic investigation into the relationship between crystal structure and NLO properties. Given the structural flexibility of perovskites and the ease of SC preparation, future efforts should prioritize the synthesis of large, high-quality SCs with broad transmission bandwidths, enhanced structural distortion, and reduced centrosymmetry to develop high-performance NLO crystals for commercial laser applications.

## Figures and Tables

**Table 1:** Summary of different synthesis methods.

Materials	Synthesis Methods	Solvents	Temperature	Size of Crystals
(MPEA)_1.5_PbBr_3.5_(DMSO)_0.5_ SCs [38]	AVC	DMF and DMSO	Room temperature	Millimeter size
(*R*/*S*)-*α*-(PEA)_2_PbI_4_ SCs [47]	Aqueous synthesis	Deionized water	Room temperature	Millimeter size
(*R*/*S*-*α*-PEA)_4_Bi_2_I_10_ SCs [48]	Temperature-lowering	55–57% HI	70 to 35 °C	1.1 cm
(*R*- and *S*-*α*-PEA)PbI_3_ SCs [36]	Temperature-lowering	45% HI	90 to 40 °C	3 mm × 2 mm × 1 mm
(*R*-/*S*-/rac-MBA)_3_InCl_6_ SCs [49]	Slow evaporation	HCl	Room temperature	1.5 mm × 0.6 mm × 0.4 mm
MAPbBr_3_ SCs [50]	Inverse temperature	DMF	60–90 °C	5–10 mm
[(*R*/*S*)-3APr]PbI_4_ SCTFs [51]	Nucleation-controlled	55–57% HI	90 °C	>1 cm
(*R*/*S*-PyEA)Pb_2_Br_6_ microwire arrays [52]	Capillary-bridge confined assembly	DMSO	70 °C	4 inch
*R*/*S*-VPEA and *R*/*S*-EPEA SCTFs [53]	In situ crosslinking polymerization	DMF/GBL	70 °C	Exceeding 100 mm^2^
CsPb(I/Br)_3_ NC [35]	Post-synthetic ligand exchange	*n*-hexane	100 °C	~100 nm
CsPbBr_3_ NCs [54]	Single-step ultrasonication	1-octadecene	N/A	~20 nm
CsPbX_3_ NCs (X = Cl, Br, and I) [55]	Supramolecular self-assembly	1-octadecene	N/A	10–15 nm

## Data Availability

No new data were created or analyzed in this study.
